# Case report: The evolving phenotype of *ESCO2* spectrum disorder in a 15-year-old Malaysian child

**DOI:** 10.3389/fgene.2023.1286489

**Published:** 2024-01-15

**Authors:** Sok-Kun Tae, Mazlan RA, Meow-Keong Thong

**Affiliations:** ^1^ Genetics and Metabolism Unit, Department of Paediatrics, Faculty of Medicine, Kuala Lumpur, Malaysia; ^2^ Genetic Medicine Unit, University of Malaya Medical Centre, Kuala Lumpur, Malaysia

**Keywords:** Roberts syndrome, *ESCO2* spectrum disorders, cohesinopathy, *ESCO2* gene, Malaysia

## Abstract

*ESCO2* spectrum disorder is an autosomal recessive developmental disorder characterized by growth retardation, symmetrical mesomelic limb malformation, and distinctive facies with microcephaly, with a wide phenotypic continuum that ranges from Roberts syndrome (MIM #268300) at the severe end to SC phocomelia (MIM #269000) at the milder end. *ESCO2* encodes a 601-amino acid protein belonging to the Eco1/Ctf7 family of acetyltransferases that is involved in the establishment of sister chromatid cohesion, which is essential for accurate chromosome segregation and genomic stability and thus belongs to a group of disorders called “cohesinopathies”. We describe a 15-year-old Malaysian female who presented with the characteristic triad of *ESCO2* spectrum disorder, with an equivocal chromosomal breakage study and normal karyotyping findings. She was initially suspected to have mosaic Fanconi anemia but whole exome sequencing (WES) showed a likely pathogenic homozygous splice variant c.955 + 2_955+5del in the *ESCO2* gene. During the 15-year diagnostic odyssey, she developed type 2 diabetes mellitus, primary ovarian insufficiency, increased optic cup-to-disc ratio with tortuous vessels bilaterally, and an evolving but distinct facial and skin hypopigmentation phenotype. Of note, there was an absence of learning disabilities. Our findings provide further evidence for *ESCO2* spectrum disorder in an Asian child and contribute to defining the clinical and radiographic spectrum.

## Introduction

*ESCO2* spectrum disorder is a rare, autosomal recessive, genetic disorder that has a broad range of clinical phenotypes from a severe type known as Roberts syndrome to a milder type known as SC phocomelia (Vega et al., 2006). Roberts syndrome (RBS, MIM #268300) was first described in 1919 in a baby boy with tetraphocomelia and cleft lip/palate (Roberts, 1919). Vega et al. (2010) established the clinical criteria for RBS, which were based on a cohort of 49 patients: growth retardation, symmetric mesomelic shortening of the limbs predominantly affecting the upper limbs, and characteristic facies with microcephaly (Vega et al., 2010). Fifty years later, Herrmann et al. reported a milder form that was described as pseudothalidomide syndrome or SC phocomelia (MIM #269000) (Herrmann et al., 1969). The prevalence of *ESCO2* spectrum disorder is unclear. Approximately 150 individuals of diverse ethnic backgrounds have been reported in the literature (Vega et al., 2006). The clinical description of this condition is rarely reported in patients of Asian ancestry. Kantaputra et al. first reported two siblings of the Lisu tribe from Thailand who were initially diagnosed with Juberg-Hayward syndrome with *ESCO2* mutations (Kantaputra et al., 2020).

*ESCO2* spectrum disorder belongs to a group of developmental disorders termed “cohesinopathies,” which are associated with biallelic pathogenic variants in the *ESCO2* gene (MIM 609353) on chromosome 8p21.1, resulting in complete or partial loss of the acetyltransferase domain (Zakari et al., 2015). Cohesin consists of a ring-shaped multiprotein complex with four subunits (SMC1A, SMC3, RAD21, and STAG1/2) and, together with regulatory factors, is essential for sister chromatid cohesion, genome organization, gene expression regulation, DNA repair, and genome safeguarding (Cucco and Musio, 2016). ESCO2 is one of the regulatory factors in the Cohesin pathway; it encodes a 601-amino acid protein belonging to the Eco1/Ctf7 family of acetyltransferases that is involved in the establishment of sister chromatid cohesion (Faramarz et al., 2020). The cohesion of sister chromatids is essential for accurate chromosome segregation and genomic stability (Tomkins et al., 1979). A cytogenetic study of RBS revealed a rod-like chromosome morphology, resulting in a “railroad-track” appearance and characteristic premature chromatid separation (PCS), otherwise known as heterochromatin repulsion (HR) or puffing (Vega et al., 2005).

A diagnosis of *ESCO2* spectrum disorder is established when there are suggestive clinical findings and biallelic pathogenic (or likely pathogenic) variants in the *ESCO2* gene as determined by molecular genetic testing, or premature chromatid separation (PCS) as determined by cytogenetic testing. In an analysis of 49 patients with *ESCO2* mutations, including 18 previously reported cases, Vega et al. reported no clear genotype/phenotype correlation (Vega et al., 2010). Many patients with *ESCO2* spectrum disorder are clinically diagnosed with many overlapping syndromes. Here, we describe a 15-year-old Malaysian female who presented with the characteristic triad of RBS, an equivocal chromosomal breakage study, and normal karyotyping, who was initially diagnosed as mosaic FA for 11 years. The diagnosis was established via whole exome sequencing (WES), where a homozygous, likely pathogenic, variant was identified in the *ESCO2* gene.

## Case description

**FIGURE 1:**
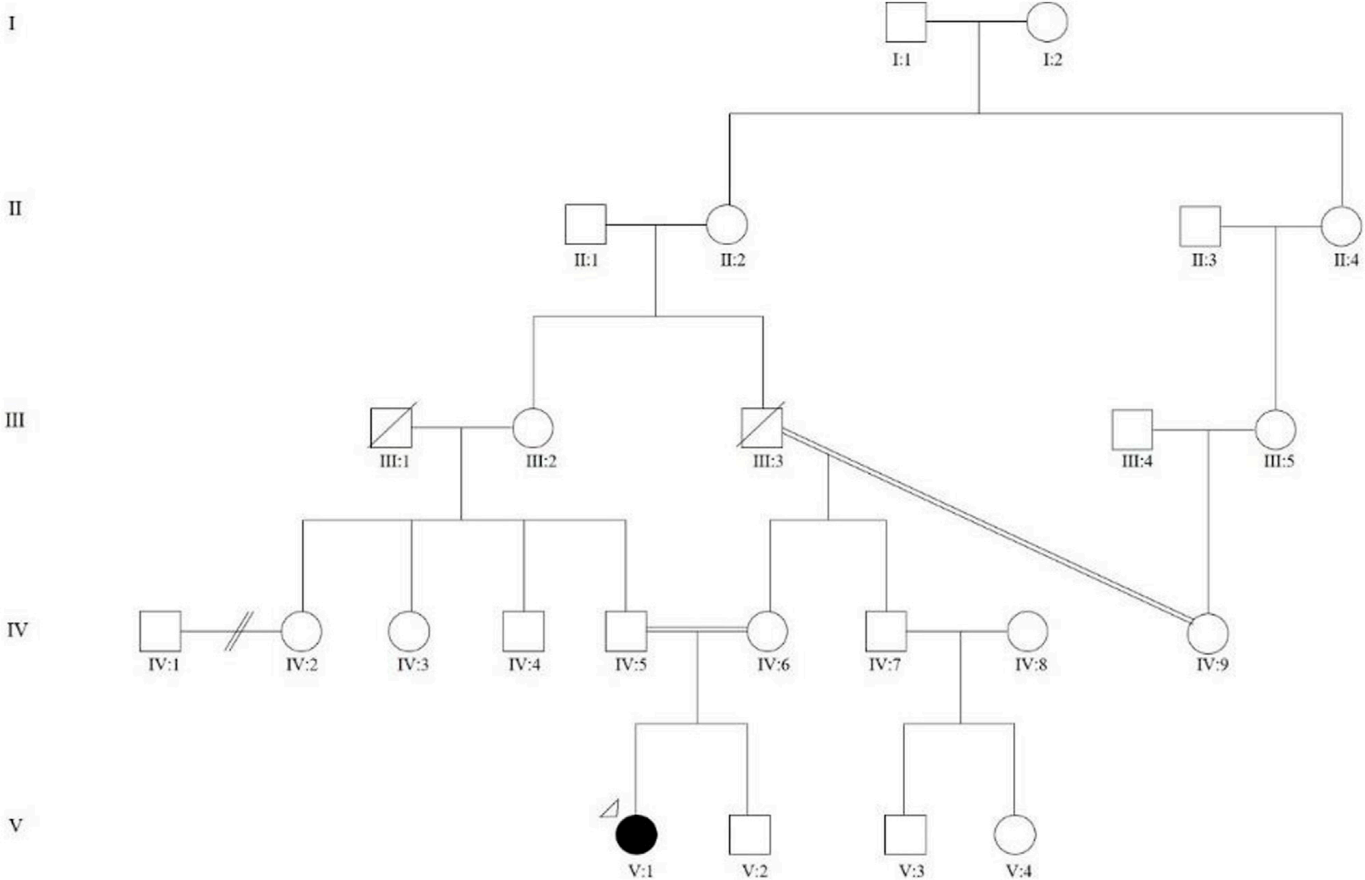
Family pedigree showing multi-level consanguinity. V:1 is the proband who was the first child of a consanguineous marriage, where her parents were first cousins (IV:4 and IV:5). The maternal grandparents (III:3 and IV:9) were third cousins.

A 15-year-old female of Indian ancestry was first referred to the Genetics and Metabolism Unit, University of Malaya Medical Centre (UMMC) at birth for multiple skeletal abnormalities and intrauterine growth retardation (IUGR), with distinctive facial features. She was the first child (V:1, see Figure 1) of a consanguineous union, where her parents were first cousins. Her mother and father were 24 and 28 years old, respectively, when she was born. The maternal grandparents were third cousins. She had a younger brother (V:2) who was healthy, with no skeletal abnormality. There was a family history of type 2 diabetes mellitus. Antenatally, her mother had gestational diabetes mellitus with good glycemic control and there was no known teratogenic exposure. Serial antenatal ultrasound scans noted oligohydramnios and intrauterine growth retardation. There was no reduced fetal movement. She was delivered at term via elective lower segment cesarean section for an abnormal lie, with Apgar scores of 7 and 9 at 1 min and 5 min, respectively. She was admitted to the neonatal intensive care unit (NICU) for 2 weeks for weight management.

The patient’s gross and fine motor developmental milestones were delayed due to the skeletal deformities. However, she had normal intellectual development and attended normal school. She was average in her school performance. She underwent multiple corrective surgeries for her lower limbs. For the bilateral congenital talipes equinovarus (CTEV), she was treated with serial casting from birth, followed by a Dennis Brown (DB) splint and shoes until the age of 7 months old. For the fixed flexion deformity of her knees, she underwent bilateral Ilizarov external fixation twice at the age of 3 and 10 years old. She had bilateral distal femur and proximal tibia hemi-epiphysiodesis performed at 6 years of age. These surgeries enabled her to walk for short distances of approximately 50–100 m; however, she developed pain and fatigue while walking for longer distances. Ophthalmology review at birth revealed an increased optic cup-to-disc ratio (CDR) at 0.8, with tortuous vessels bilaterally. There was no increase in intra-ocular pressure, no lens or corneal opacities, nor retinal abnormalities.

**FIGURE 2:**
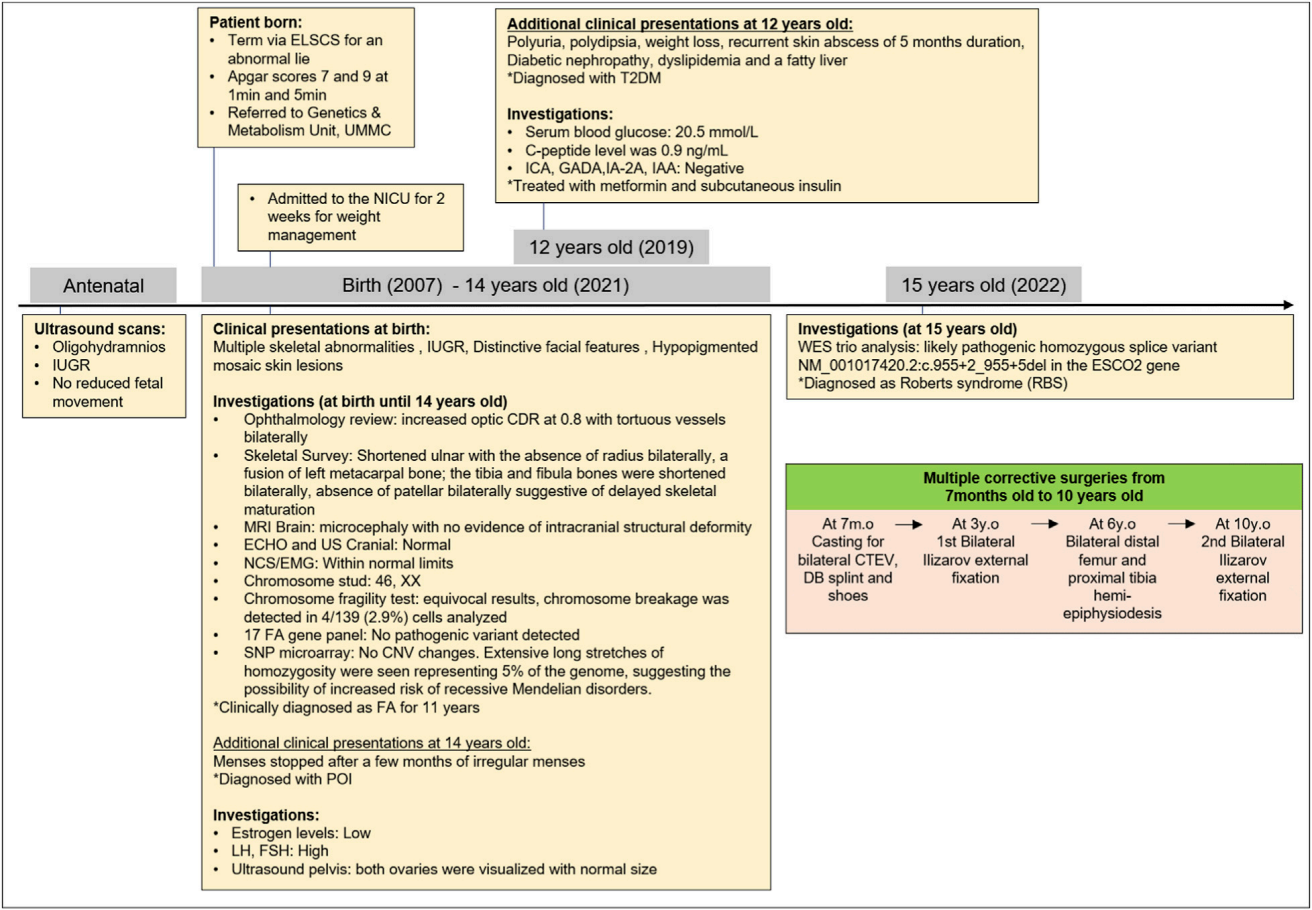
Clinical course and diagnostic timeline. *ELCCS, emergency lower C-section; UMMC, University Malaya Medical Centre; NICU, Neonatal Intensive Care Unit; IUGR, intrauterine growth restriction; T2DM, type 2 diabetes mellitus; ICA, islet cell cytoplasmic autoantibodies; GADA, glutamic acid decarboxylase autoantibodies; IA-2A, insulinoma-associated-2 autoantibodies; IAA, insulin autoantibodies; LH, high luteinizing hormone; FH, follicle-stimulating hormone; CDR, cup-to-disc ratio; MRI: magnetic resonance imaging; ECHO, echocardiogram; US, ultrasound; NCS, nerve conduction study; EMG, electromyography; FA, Fanconi anemia; SNP: CNV, copy number of variant; POI, primary ovarian insufficiency; WES, whole exome sequencing; DB, Dennis Brown; CTEV, congenital talipes equino varus; y.o, years old; m.o, month old.

She was diagnosed with type 2 diabetes mellitus (T2DM) at the age of 12 years, where she presented with polyuria, polydipsia, significant weight loss, and recurrent skin abscess for a duration of 5 months. The diagnosis was confirmed by a serum blood glucose level of 20.5 mmol/L, with no metabolic acidosis or ketosis and a c-peptide level of 0.9 ng/mL. There were negative islet cell cytoplasmic autoantibodies (ICA), glutamic acid decarboxylase autoantibodies (GADA), insulinoma-associated-2 autoantibodies (IA-2A), and insulin autoantibodies (IAA). She was treated with metformin and subcutaneous insulin. Her T2DM was complicated by diabetic nephropathy, dyslipidemia, and a fatty liver. She attained menarche at 14 years old and was diagnosed with primary ovarian insufficiency when her menses stopped after a few months of irregular menses. A hormonal study revealed low estrogen levels, with high luteinizing hormone (LH) and follicle-stimulating hormone (FSH) levels. Pelvis ultrasonography visualized both ovaries, which were normal in size. Figure 2 shows the clinical course and diagnostic timeline for 15 years.

**FIGURE 3:**
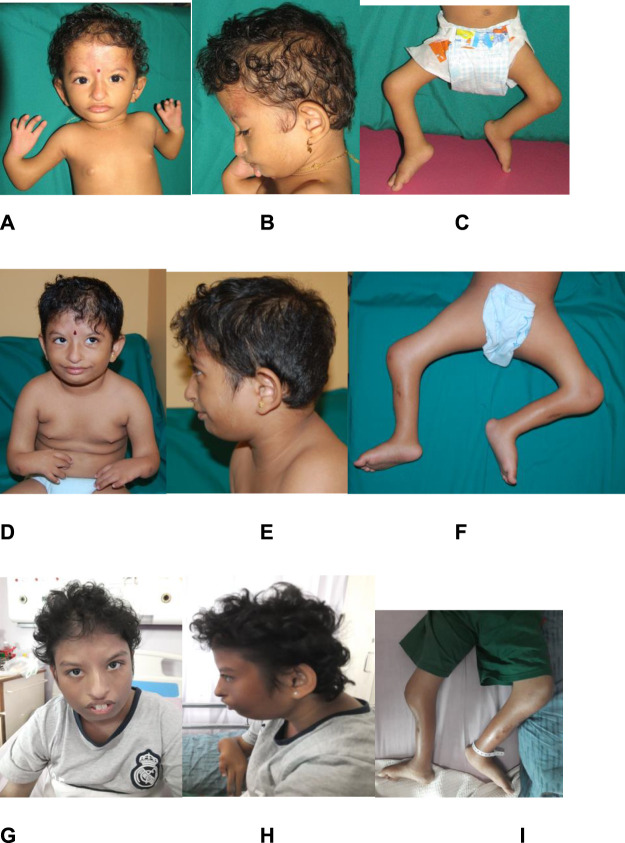
Evolving phenotype of the patient over 15 years. Photographs of the patient at 2 years old (images **(A–C)**), at 6 years old (images **(D–F)**), and at 14 years old (images **(G–I)**). Images **(A,B)**, **(D,E)**, and **(G,H)** show diffuse alopecia, brachymicrocephaly, down-slanting of palpebral fissures, hypertelorism, hypoplastic nasal alae, bilateral low-set and simple ear, malar hypoplasia, and micrognathia. Images **(C,F,I)** show fixed flexion deformities of both her knees.

Physical examination at birth showed prenatal growth failure, with a birth weight of 1.46 kg (Z = −4.7 SD), length of 35 cm (Z = −8.1 SD), and head circumference of 26 cm (Z = −8.0 SD). Distinctive features included brachymicrocephaly, diffuse alopecia, down-slanting of palpebral fissures, hypertelorism, hypoplastic nasal alae, bilateral low-set and simple ears, malar hypoplasia, and micrognathia (Figure 3). There was no cleft or high-arched palate. Bilateral symmetrical mesomelic shortening of the upper and lower limbs was found, where the upper limbs were more severely affected. There were bilateral absent radii, an absent left thumb with a small, rudimentary right thumb, and bilateral congenital talipes equinovarus. Capillary hemangioma was present at birth over the forehead and extending down to the nose, which gradually faded away when she was a toddler. There were hypopigmented mosaic skin lesions noted since birth over her left cheek, chin, upper limbs, abdomen, and back, which followed Blaschko’s lines. These lesions became more obvious and prominent over time. At 15 years of age, all her growth parameters remained well below the third centile.

**FIGURE 4:**
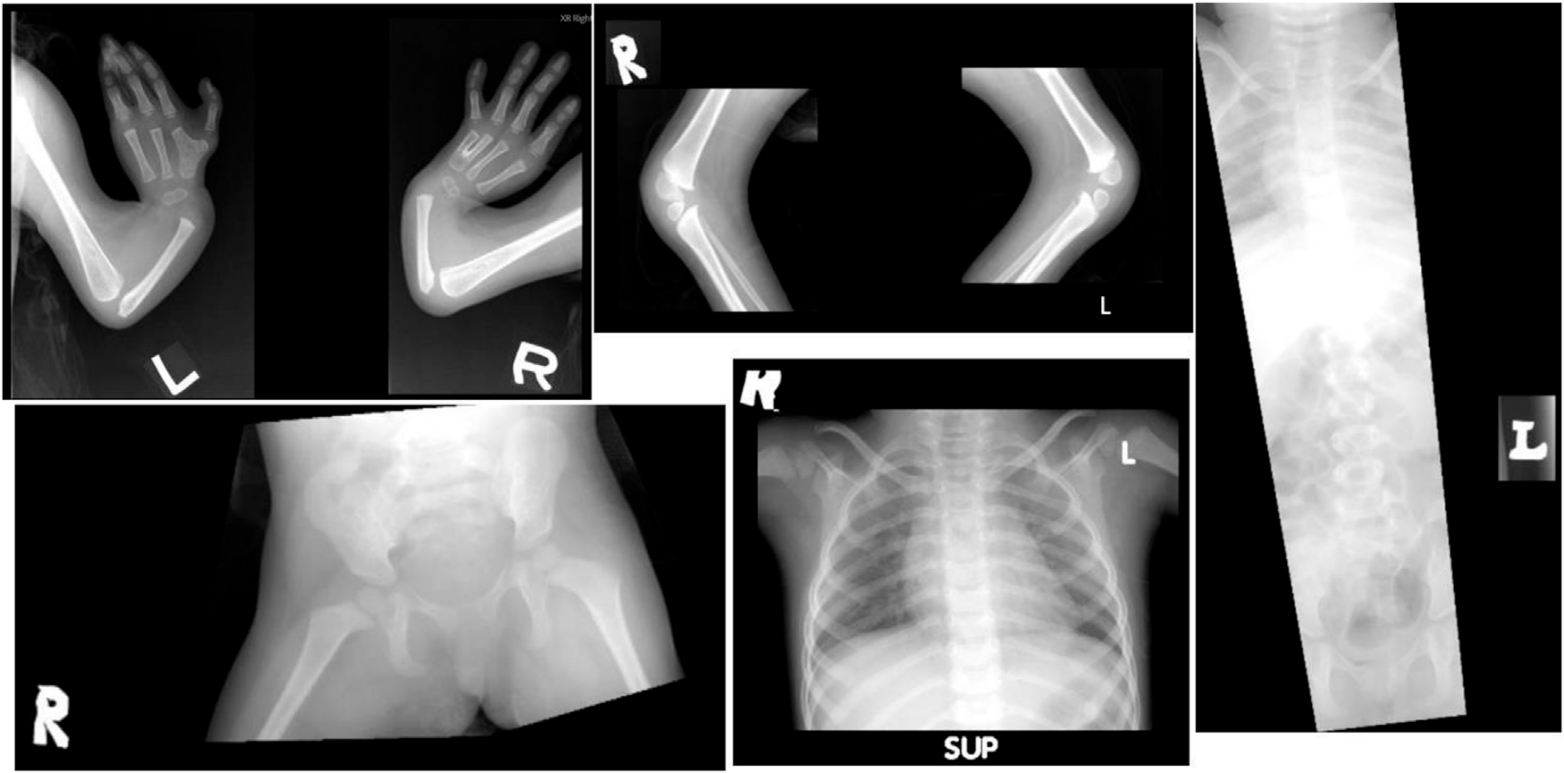
Patient’s radiograph at the age of 2 years old. There was a shortened ulnar, with the absence of radius bilaterally, and fusion of the left metacarpal bone. The spine and pelvic bone appeared normal.

Based on her clinical features, a diagnosis of Fanconi anemia (FA) was made. A skeletal survey performed at birth showed a small skull with micrognathia, short ulnae with the absence of radii bilaterally, fusion of the left metacarpal bones, short tibiae and fibulae bones bilaterally, and the absence of patellar bilaterally, suggestive of delayed skeletal maturation. The spine and pelvic bone appeared normal (Figure 4). Cranial MRI showed microcephaly, with no evidence of intracranial structural abnormalities. The echocardiogram was normal. Given multiple contractures, a nerve conduction study and electromyogram were performed, and both were within normal limits. A chromosome study revealed normal results, 46, XX. A chromosome fragility test showed equivocal results, where chromosome breakage was detected in 4/139 (2.9%) cells analyzed. Serial complete blood count showed no evidence of bone marrow failure. A multigene panel sequencing of 17 FA genes showed no pathogenic variant detected, while a chromosomal SNP microarray revealed no clinically significant copy number changes. However, extensive long stretches of homozygosity were seen, representing 5% of the genome, suggesting the possibility of an increased risk of recessive Mendelian disorders.

The parents were devastated by the negative results. However, they were motivated to pursue further investigations. The patient was treated as mosaic FA and medical surveillance was continued.

The patient was included in the Global Genomic Medicine Collaborative (G2MC) Rare Disease project, in which whole exome sequencing detected a homozygous splice variant NC_000008.10(NM_001017420.3): c.955 + 2_955+5del (GRCh37 (chr8): g.27637786_27637789del) in the *ESCO2* gene in the patient. This variant does not present nucleotide change as it is an intronic splicing variant. The frequency allele in gnomAD v2.1.1 is 0.000012 and in the South Asian population is 0.00009827. This sequence change affects a splice site in intron 4 of the *ESCO2* gene. It is predicted to alter splicing and results in loss of protein function. Loss of function variants in *ESCO2* are known to be pathogenic (Schüle et al., 2005; Vega et al., 2005). Both her parents were heterozygous carriers. This variant was close to the highly conserved splice site. According to HGMD Professional 2021.3, this variant was previously described as disease-causing for Roberts syndrome (Gordillo et al., 2008; Capalbo et al., 2019). ClinVar listed this variant as likely pathogenic (Variation ID: 21252), and homozygosity was confirmed by parental testing. According to the recommendations of the ACMG/AMP variant classification guidelines (Richards et al., 2015), this variant was classified as likely pathogenic.

## Discussion

To the best of our knowledge, this is the first case report of an individual with *ESCO2* spectrum disorder/RBS with Asian Indian ancestry with the classical triad of RBS that consisted of pre-and post-natal growth retardation, bilateral symmetrical mesomelic limb shortening predominantly affecting her upper limbs, and distinctive facial features with microcephaly (Vega et al., 2010). Her limb malformations include bilateral short ulnar and absent radii, thumb defects, oligodactyly, left metacarpal synostosis, flexion contractures over the elbows and knees, bilateral short tibia and fibula, absent patella, and bilateral CTEV. The limb deformities noted in our patient that are rarely reported were flexion contractures (16%), metacarpal synostosis (12%), and absent patella (4%) (Vega et al., 2010). The unique aspect of our case is the 15-year follow-up of the patient’s progress. Whilst she had gross and fine motor impairment due to her skeletal deformities, there was no intellectual impairment. This included the documentation of the absence of learning disabilities and a non-progressive increase in CDR. Interestingly, our patient developed T2DM and primary ovarian insufficiency (POI), which were not reported before in other patient series. The etiology of POI could be autoimmune (4%–30%) (Kirshenbaum and Orvieto, 2019) or genetic (França and Mendonca, 2019), although the majority remains unknown. A few case reports showed a biallelic pathogenic variant in *STAG3*, a cohesion gene, affected the meiosis-specific cohesion complexes, which might lead to premature ovarian failure (Caburet et al., 2014; Colombo et al., 2017; Akbari et al., 2022). Other meiosis-specific complexes in cohesin, including REC8, RAD21L, and SMC1β, have been shown to be associated with abnormal oocyte development (Caburet et al., 2014; Ward et al., 2016). Thus, more studies will be required to investigate the relationship between the *ESCO2* gene, regulatory factors in the cohesin pathway, and human fertility. The evolving facial phenotype and hypopigmented skin lesions documented in our patient will serve as a potential guide for counseling and future dysmorphological studies in the Asian population for RBS/*ESCO2* spectrum disorders.

Our patient was initially diagnosed as having FA for 11 years. Some of the clinical characteristics, namely, skeletal deformities, growth retardation, microcephaly, and the extent of MMC-induced chromosomal breakage in patients with cohesinopathies overlapped with those of (mosaic) FA patients (Lo Ten Foe et al., 1997). Patients with mosaic FA may present without bone marrow failure due to spontaneous genetic reversion, which corrects the bone marrow failure (van der Lelij et al., 2010). Chromosomal breakage study, which is the gold standard in diagnosing FA (Hirsch et al., 2020), could be equivocal in both patients with mosaic FA and cohesinopathies. Chromosomal breakage study on skin fibroblasts or conventional karyotyping showed “railroad-track” chromosomes and premature chromatid separation (PCS), which allowed these two conditions to be distinguished. In our patient, the basic karyotyping performed earlier showed normal results, and a chromosomal breakage study on skin fibroblasts was not performed. Due to the highly variable clinical phenotype, a patient with a cohesinopathy such as RBS may exhibit multiple clinical features that overlap with mosaic FA. Cellular hypersensitivity to MMC has also been reported in cohesinopathies, including RBS (van der Lelij et al., 2010). Thus, our patient was treated as mosaic FA initially but the diagnostic odyssey ended with the whole exome sequencing (WES) test, which demonstrated the homozygous splice site variant c.955 + 2_955+5del in the *ESCO2* gene. It was important to establish an accurate diagnosis as there were implications for clinical management and surveillance as well as for genetic counseling. When she was initially diagnosed as having Fanconi anemia, the family was informed of an increased risk of tumors, and she received annual ultrasonography of the liver and blood tests such as complete blood count and liver function tests. With the revised diagnosis of *ESCO2* disorder, frequent blood tests and liver ultrasonography were no longer required as the cancer risk was not increased. In addition, as both parents were carriers, the family was counseled on the autosomal recessive inheritance and recurrence risks. Cascade screening for extended family members was also performed.

The protracted journey toward the final diagnosis for people living with rare diseases, where up to 50% will remain undiagnosed (Shashi et al., 2014), is not surprising. The clinical implementation of WES has helped in shortening the odyssey and has had significant clinical, genetic counseling, psychosocial, and economic benefits (Sawyer et al., 2016). It was reported that WES had a positive diagnostic yield of approximately 39% in diagnosing disorders related to skeletal malformations (Retterer et al., 2016). There is a need to assess the clinical utility and cost-effectiveness of WES in ending the diagnostic odyssey in low-resourced countries for Asian children with dysmorphic features and multiple congenital malformations. Further discussion with all stakeholders, patient support groups, and governmental intervention is important to ensure equity of care and an appropriate funding model for patients with rare diseases.

## Data Availability

The datasets for this article are not publicly available due to concerns regarding participant/patient anonymity. Requests to access the datasets should be directed to the corresponding author.
